# ^**1**^H and ^**13**^C NMR Assignments of Cytotoxic 3*S*-1,2,3,4-Tetrahydro-****β****-carboline-3-carboxylic Acid from the Leaves of *Cichorium endivia*

**DOI:** 10.1155/2012/254391

**Published:** 2012-12-16

**Authors:** Fu-Xin Wang, An-Jun Deng, Jin-Feng Wei, Hai-Lin Qin, Ai-Ping Wang

**Affiliations:** Institute of Materia Medica, Chinese Academy of Medical Sciences and Peking Union Medical College, Beijing 100050, China

## Abstract

An amino acid, 3*S*-1,2,3,4-tetrahydro-**β**-carboline-3-carboxylic acid, was isolated for the first time from the leaves of *Cichorium endivia*. The complete assignment of its ^1^H and ^13^C NMR spectroscopic data was carried out also for the first time based on extensive 1D and 2D NMR experiments. Cytotoxicity of this isolated compound against HCT-8 and HepG2 human cancer cell lines was evaluated for the first time, with moderate activities being found.

## 1. Introduction

*Cichorium endivia* L. is a popular vegetable from the family of Compositae and is widely cultivated and consumed all over the world. Its popularity is also attributed to the healthy properties mainly due to supply of antioxidant activity [1, 2]. However, phytochemical investigation on this plant is very rare up to now, to the best of our knowledge, only a few papers had reported a few compounds, including five ones by our group [3]. The ongoing research aims at confirming the bioactive compounds from this popular vegetable, and a prevailing and known amino acid, 3*S*-1,2,3,4-tetrahydro-**β**-carboline-3-carboxylic acid (**1**), was isolated for the first time. By way of the literature survey, it can be learned that the complete assignment of the NMR data of **1** was very deficient up to now, with no practical conducting being obtained due to the poor solubility of **1** in most prevalent solvents and causing the citation of literatures an obvious state of chaos [4, 5]. In this paper, we describe the complete assignment of the ^1^H and ^13^C NMR spectroscopic data of **1** based on the determining of optimized solvent and extensive 1D and 2D NMR experiments. An investigation focusing on the cytotoxicity of compound **1** against HCT-8 and HepG2 human cancer cell lines showed that **1** inhibits the cells growth by a moderate reduction in viability of subjects.

## 2. Results and Discussion

**Figure 1:**
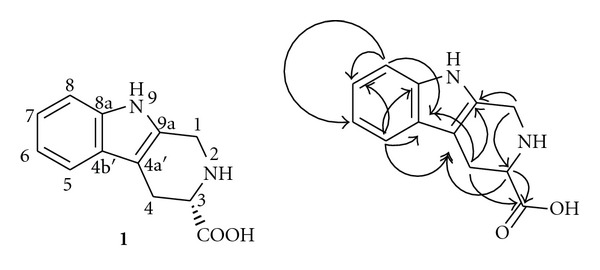
The structure of compound **1** and the key HMBC (→ : H→C) correlations.

Compound **1** was isolated as an amorphous pale-yellow powder (MeOH/H_2_O). Its positive-ion ESI-MS spectrum showed the quasimolecular ion peaks at m/z 217.1 [M+H]^+^and 239.1 [M+Na]^+^, and its molecular formula was established to be C_12_H_12_N_2_O_2_ by the quasimolecular ion peak in the positive mode HRESI-MS experiment at m/z 217.0967 [M+H]^+^. The IR spectrum showed strong absorptions at *υ*_max⁡_ 3284, 3019, 1642, 1598, 1452, 1409, and 740 cm^−1^, indicating the presence of 1,2-disubstitued benzene moiety and labile hydrogen. It was preliminarily identified as 3*S*-1,2,3,4-tetrahydro-**β**-carboline-3-carboxylic acid by comparison of the ^1^H NMR spectroscopic data obtained in DMSO-d_6_ with the literature values [4, 5], but some obvious errors or inconsistency were evident, including the coupling constants and data ownership (Table 1). Whereas the obtainment of ^13^C NMR spectrum in the NMR solvent of DMSO-d_6_ was very difficult due to the above-mentioned poor solubility, compound **1** was then recorded the 1D and 2D NMR spectra within D_2_O + drops of F_3_CCOOD, which proved to be a good solvent for **1**. The ^1^H NMR spectroscopic data also clearly revealed the existence of 1,2-disubstitued benzene moiety, with four diagnostic signals from an aromatic ABCD spin system resonating at *δ*_H_ 7.13 (1H, t, *J* = 7.6 Hz, H-6), 7.22 (1H, t, *J* = 8.0 Hz, H-7), 7.43 (1H, d, *J* = 8.0 Hz, H-8), and 7.53 (1H, d, *J* = 8.0 Hz, H-5), which correlated to the aromatic carbon signals at *δ*_C_ 120.0 (C-6), 122.9 (C-7), 111.9 (C-8), and 118.2 (C-5), respectively, in the HSQC spectrum. In addition, five well-resolved and characteristic signals at *δ*_H_ 4.38 (1H, d, *J* = 15.6 Hz, H-1_a_), 4.54 (1H, d, *J* = 15.6 Hz, H-1_b_), 4.27 (1H, dd, *J* = 10.4, 5.6 Hz, H-3), 3.13 (1H, dd, *J* = 10.8, 16.4 Hz, H-4_a_), and 3.38 (1H, dd, *J* = 5.6, 16.4 Hz, H-4_b_) were also examined in the ^1^H NMR spectrum, which were, conveniently according to their coupling constants and with the aid of ^1^H, ^1^H-COSY spectrum, assigned to one AB spin system from an isolated methylene group and one ABX system from one methylene and one methine correlated together by *sp* ^3^ hybridized bond. These three functional groups were obviously deshielded and were correlated to their corresponding carbon signals at *δ*_C_ 40.5 (C-1), 55.0 (C-3), and 21.7 (C-4), respectively, in the HSQC spectrum. Simply, these three groups were arranged to either *sp* ^2^ hybridized carbons or nitrogen atoms when considering the ^1^H and ^13^C NMR chemical shifts and examining the ^13^C and DEPT NMR data which exhibited twelve carbon signals with two aliphatic methylenes, one aliphatic methine, four *sp* ^2^ hybridized methines, and five *sp* ^2^ hybridized quaternary carbons being categorized (Table 1). The above NMR data were compatible with a benzene moiety, a tetrasubstituted ethylene, and a carbonyl except for the above-mentioned three aliphatic carbons. The long range ^1^H,^13^C-correlations from *δ*_H_ 7.53 to *δ*_C_ 104.9, 125.6, 136.7, and 122.9, from *δ*_H_ 7.43 to *δ*_C_ 122.9, 120.0, and 125.6, from *δ*_H_ 7.22 to *δ*_C_ 111.9, 136.7, and 118.2, from *δ*_H_ 7.13 to *δ*_C_ 118.2, 125.6, 122.9, and 111.9, from *δ*_H_ 4.38 and 4.54 to *δ*_C_ 125.4, 104.9, and 55.0, from *δ*_H_ 3.13 and 3.20 to *δ*_C_ 104.9, 55.0, 171.3, and 125.6/125.4, and from *δ*_H_ 4.27 to *δ*_C_ 171.3, 40.5, 21.7, and 104.9 established the constitutional formula of **1 **as indicated by Figure 1. The complete assignment of the NMR data is listed in Table 1.

On evaluation of compound **1** for its cytotoxic effects on two human cancer cell lines, cell growth was measured using a sulforhodamine B (SRB) assay. Results of means of three replicates are expressed as the percentage of viability compared to negative control. Compound **1** exhibited moderate cytotoxicities against HCT-8 and HepG2 cell-lines in the evaluation, with viability of HepG2 and HCT-8 cells being 80.42% and 80.22% after treatment for 48 hours, and 76.14% and 71.48% after 72 hours, respectively, when using a concentration of 140 *μ*g/mL. The viabilities at other time points were relatively lower.

## 3. Experimental

### 3.1. General Experimental Procedures

IR spectra were obtained on a Nicolet 5700 spectrometer. 1D and 2D NMR spectra were recorded on a Mercury-400 or a MERCURY-300 NMR spectrometer. Chemical shifts (*δ*) were given in ppm using tetramethylsilane (TMS) as internal standard (*δ* 0.00). ESI-MS and HRESI-MS were measured on an Agilent 1100 series LC-MSD-Trap-SL spectrometer. RP-18 (YMC-GEL, ODS-A, 12 nm, S-50 mm; YMC Co., Kyoto, Japan) were used for column chromatography. Solvents were of analytical grade and were purchased from Beijing Chemical Company (Beijing, China).

### 3.2. Plant Material

*Cichorium endivia* was purchased from Beijing Xinfadi agricultural products wholesale market on July 2009 and authenticated by Associate Professor Ma Lin (Institute of Material Medica, Chinese Academy of Medical Sciences and Peking Union Medical College). A voucher specimen was deposited in New Drug Safety Evaluation Center, Institute of Materia Medica, Peking Union Medical College & Chinese Academy of Medical Sciences, China.

### 3.3. Extraction and Isolation

The air-dried and pulverized *C. endivia* (5.8 kg) was extracted three times under reflux conditions with 95% EtOH (80 L, 2 h; 70 L, 1 h; 68 L, 1 h). The combined EtOH extracts were evaporated in vacuum to yield a dark-green residue (1233 g, crude EtOH extract), which was suspended in 80% aq. EtOH. The resulting suspension was extracted with petroleum ether (60–90°C, 2 L, 1.5 L, 1.5 L, and 1.5 L). Evaporation of the aq. layers in vacuum yielded also a dark-green residue (940 g, 80% EtOH extract). The residue was redissolved in water and subsequently partitioned with EtOAc in separatory funnel exhaustively. The rest of water soluble fraction was loaded on a column filled with Daion HP-20 and eluted with H_2_O, 60% EtOH, and 95% EtOH, respectively. The eluates of 60% EtOH were evaporated in vacuum which yielded a black residue (40 g). 

The 60% EtOH fraction was redissolved in solvent of *n*-BuOH and washed with aq. 5% NaHCO_3_ then H_2_O, respectively. Evaporation of *n*-BuOH under reduced pressure gave 5.5 g of brown-green residue, which was submitted to an ODS-A column eluted with MeOH-H_2_O of decreasing polarity (40%–100%) to yield compound **1** (108 mg) as pale-yellow powder.

#### 3.3.1.  3*S*-1,2,3,4-Tetrahydro-*β*-carboline-3-carboxylic Acid (******1******)

Pale-yellow powder. IR (KBr) *υ*_max⁡_ (cm^−1^): 3284, 3019, 2849, 1642, 1598, 1452, 1409, 1271, 1221, and 740; ^1^H and^13^C NMR spectroscopic data are listed in Table 1. ESI-MS (positive mode) m/z: 217.1 [M+H]^+^and 239.1 [M+Na]^+^; HRESI-MS (positive mode) m/z: 217.0967 [M+H]^+^(calcd for C_12_H_12_N_2_O_2_, 217.0972).

### 3.4. Cytotoxicity Assay

HCT-8 and HepG2 cells were cultivated in RPMI1640 medium containing 0.22% sodium bicarbonate, 10% fetal bovine serum (FBS), 100 U/mL penicillin, and 100 *μ*g/mL streptomycin. The cells were incubated in 5% CO_2_-air at 37°C. Compound **1** was dissolved in phosphate-buffered saline (PBS) at a concentration of 1.4 mg/mL and was diluted to the required concentration with RPMI1640 medium immediately before use.

The cell viability was measured by using sulforhodamine B (SRB) assay. Briefly, the cells were seeded in 96-well plates (1 × 10^4^ cells/well) and routinely cultured for 24 h. Compound **1** was added to in-serial concentrations (from 14 *μ*g/mL to 140 *μ*g/mL), while PBS was added alone to control wells as a negative control, and incubation was continued for an additional 48 h. SRB (1 mg/mL) was added to each well after the plates were fixed using TCA (0.4% m/v). After 20 minutes of incubation, each well was washed by acid (1% v/v) three times. Then wells were added into Tris (100 mmol/L), respectively. The absorbance of each well was recorded on a microplate spectrophotometer at 515 nm.

## Figures and Tables

**Table 1:** ^
1^H and ^13^C NMR spectroscopic data for compound **1**.

Number	*δ*_H_^ab^	*δ*_C_^ac^	*δ*_H_^de^	*δ*_H_^d^ [4]	*δ*_H_^d^ [5]	*δ*_C_^d^ [5]
1a	4.38 d (15.6)	40.5 t	4.15 d (15.3)	—	—	40.3
1b	4.54 d (15.6)		4.23 d (15.9)	4.22 d (4.8)	—	
3	4.27 dd (10.4, 5.6)	55.0 d	3.60 m	3.14	—	55.3
4a	3.13 dd (10.8, 16.4)	21.7 t	2.81 dd-like	2.83 ddd (10.5, 5.0, 2.4)	—	18.0
4b	3.38 dd (5.6, 16.4)		3.13 br d-like	3.69 dd (10.5, 5.0)	—	
4a′		104.9 s				104.3
4b′		125.6^f^ s				128.5
5	7.53 d (8.0)	118.2 d	7.32 d (7.5)	7.33 d (8.0)	7.38 d (8.2)	118.5
6	7.13 t (7.6)	120.0 d	7.06 t (7.5)	7.08 t (8.0)	7.06 t (8.2)	117.5
7	7.22 t (8.0)	122.9 d	6.97 t (7.8)	6.99 t (7.5)	6.96 t (8.2)	121.1
8	7.43 d (8.0)	111.9 d	7.43 d (7.5)	7.44 d (7.5)	7.44 d (8.2)	111.8
8a		136.7 s				136.1
9a		125.4^f^ s				—
COOH		171.3 s	10.91 s	10.93 s	—	165.6
9-NH				—	10.66 s	

^a^D_2_O + drops of F_3_CCOOD; ^b^400 MHz. ^c^100 MHz. ^d^in DMSO-d_6_; ^e^300 MHz. ^f^Assignments may be interchanged.
